# Ion correlations explain kinetic selectivity in diffusion-limited solid-state synthesis reactions

**DOI:** 10.1038/s41563-026-02596-5

**Published:** 2026-04-28

**Authors:** Vir Karan, Max C. Gallant, Yuxing Fei, Gerbrand Ceder, Kristin A. Persson

**Affiliations:** 1 Materials Sciences Division, Lawrence Berkeley National Laboratory, Berkeley, CA USA; 2 Department of Materials Science and Engineering, University of California, Berkeley, Berkeley, CA USA

**Keywords:** Atomistic models, Solid-phase synthesis, Computational chemistry

## Abstract

Establishing viable solid-state synthesis pathways for novel inorganic materials remains a major challenge in materials science. Previous pathway design methods using pairwise reaction approaches have navigated the thermodynamic landscape with first-principles data but lack kinetic information, limiting their effectiveness. This gap leads to suboptimal precursor selection and predictions, especially for reactions forming competing phases with similar formation energies, where ion diffusion is a critical influence. Here we demonstrate an inorganic synthesis framework by incorporating machine learning-derived transport properties through ‘liquid-like’ product layers into a thermodynamic cellular reaction model. In the Ba–Ti–O system, known for its competitive polymorphism, we obtain accurate predictions of phase formation with varying BaO:TiO_2_ ratios as a function of time and temperature. We find that diffusion–thermodynamics interplay governs phase compositions, with cross-ion transport coefficients critical for predicting diffusion-limited selectivity. This work bridges length scales and timescales by integrating solid-state reaction kinetics with first-principles thermodynamics and spatial reactivity.

## Abstract (web summary)

Cross-ion correlations are crucial to quantitatively explain the appearance of intermediate phases and final product fractions during powder synthesis.

## Main

The demand for new inorganic materials to improve energy technologies has led to the emergence of powerful, data-driven materials design. However, realizing in silico-designed materials through inorganic synthesis remains a challenge, lagging behind organic synthesis due to the absence of a general mechanistic model for solid-state reactions^1^. Current state-of-the-art atomistic modelling of solid-state synthesis describes reaction behaviour in terms of bulk thermodynamic properties from high-throughput databases of density functional theory (DFT) calculations such as the Materials Project (MP)^2^. Prominent examples include reaction networks^3^ (which identify thermodynamically favourable pathways) and active learning algorithms^4^ (which iteratively refine synthesis recipes based on experimental feedback). However, predictions based solely on thermodynamics can be inaccurate, particularly in systems with competing phases of similar formation energies^5^. In such cases, the limited transport of essential constituents may prevent the formation of globally stable products, hindering the attainment of thermodynamic equilibrium. Prior attempts to understand such effects have led to the use of empirical rate expressions^6,7^, which fit ‘effective rate constants’ from the degree of conversion of the reactants, making them post hoc and not predictive of solid-state reaction products. Computational studies of these reactions largely rely on phase-field methods, which require solving coupled, multiphysics partial differential equations. Prior work has focused on simplified scenarios, including single-step reactions without intermediates^8^, sintering-driven morphology evolution^9^ and the growth of predefined nucleated phases in melts^10^. Although informative, these studies depend on experimental or modelled inputs and, thus, require well-characterized systems to resolve diffusion and nucleation kinetics^11^.

**Fig. 1:**
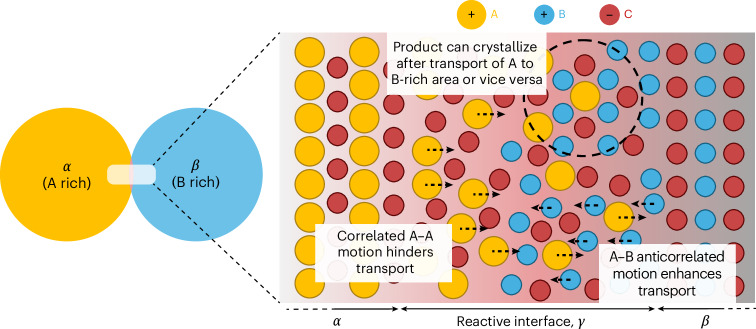
Reactive interface between two precursors, one of which is cation A rich (*α*) and the other is cation B rich (*β*), which react to form an interphase *γ*. The diffusion of A towards the B-rich precursor through the disordered/liquid-like region of the interface will determine the local availability of A and B ions, which, in turn, controls which phases can form and at what rate.

In general, solid-state reaction kinetics can be broken down into nucleation-limited and diffusion-limited regimes^1,12^. Although the former provides an insight into the first phase(s) that forms, which is useful in, for example, thin-film synthesis, such information does not conclusively predict the bulk distribution of products in powder reactions, which proceeds by the diffusion-controlled transfer of the precursor constituents to the reaction zone (Fig. 1). We hypothesize that the synthesis evolution of such systems can be described as an optimization of the local energy under the time-dependent constraint of available ionic fluxes through a defective, liquid-like interphase with the same stoichiometry as candidate, nucleating phases. Here local energy denotes the energy at a prescribed reaction interface—that is, the energy that can be reduced via the reaction of solid reactants without explicitly enforcing global mass constraints. Simply put, we propose that the growth of the crystalline nucleus in a pairwise powder reaction is governed by the ionic transport of its constituents through a liquid-like interphase of the same composition as the nucleus.

**Fig. 2:**
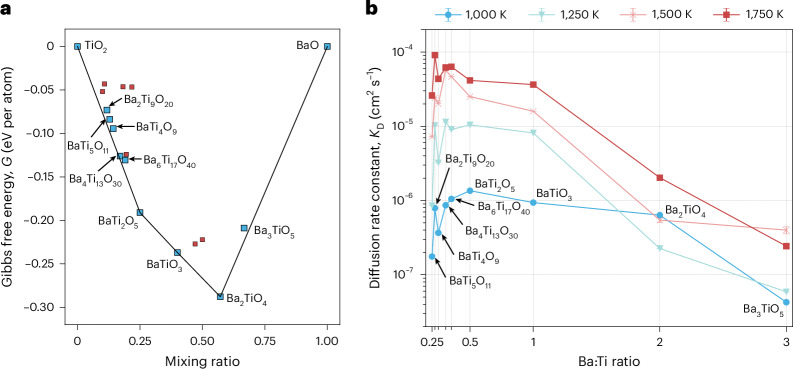
Thermodynamic and kinetic landscape in the Ba–Ti–O chemical space. **a**, Thermodynamic hull of stability for BaO and TiO_2_ at 600 °C, computed using entries from the MP. Finite-temperature effects are accounted for using a machine learning estimator for the vibrational contribution to entropy^37^. The blue squares (except Ba_3_TiO_5_) are phases that have been experimentally observed in solid-state reactions, and the red ones are additional phases in the MP. The shape of the hull (that is, the phases and their relative formation energies) remains unchanged for all the temperatures considered in this work. **b**, Calculated effective diffusion rate constant (*K*_D_), which is a measure for the average flux of all ions through the product phase of a reaction, across the BaO–TiO_2_ reaction interface at a series of temperatures in the range of 1,000–1,750 K, ordered by increasing Ba:Ti ratio. *K*_D_ peaks for phases with intermediate Ba:Ti ratio, and drops substantially in phases with Ba:Ti > 1 at typical synthesis temperatures. Error bars, where visible, in *K*_D_ are computed using the standard deviation across five MD trajectories. Source data

To showcase our model, we choose the Ba–Ti–O chemical space, which exhibits particularly competitive polymorphism (Fig. 2a). Technologically relevant Ba–Ti–O ternaries (for example, BaTiO_3_, Ba_2_Ti_9_O_20_, BaTi_5_O_11_ and BaTi_2_O_5_) are typically synthesized from BaCO_3_ and TiO_2_ precursors at varying ratios. The formation of ferroelectric BaTiO_3_ is well studied^13,14^, commonly via heating mixed powders at 1,000–1,300 °C. Although BaTiO_3_ is the primary product, Ba_2_TiO_4_ is energetically favoured at the TiO_2_/BaO interface (Fig. 2a). Impurities depend on synthesis conditions, with Ba_2_TiO_4_ dominant below 1,050 K and higher temperatures (~1,200 K) increasing the BaTiO_3_ yield but promoting secondary phases such as BaTi_2_O_5_.

**Fig. 3:**
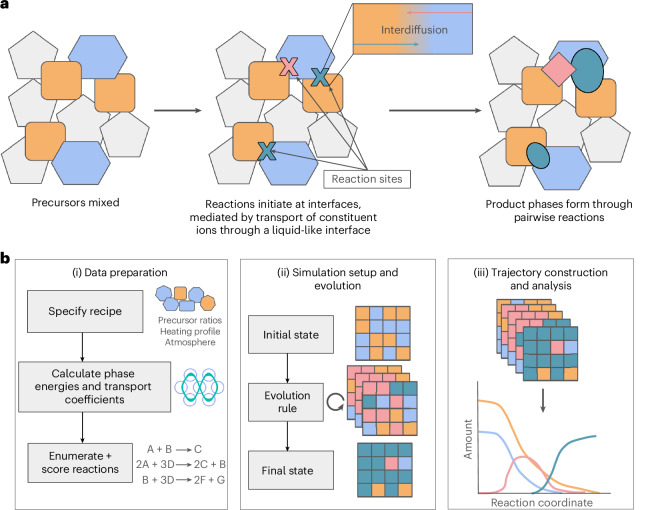
Overview of ReactCA simulation framework for simulating solid-state powder synthesis reactions. **a**, Progression of one step of the reaction via pairwise interfacial reactions. **b**, Schematic illustrating the main stages of the framework: (i) the user specifies synthesis recipe to study (consisting of the chemical system to be considered, precursors and their amounts, reaction atmosphere and heating profile), based on which formation energies are obtained from the MP and machine learning estimators are used to compute the vibrational free energies, melting points and diffusion rate constants, and reactions are then enumerated and scored; (ii) a random initial distribution of particles (voxels) is generated, and the evolution rule is repeatedly applied using the scores assigned to each reaction to simulate the reaction; (iii) simulation steps are concatenated into a trajectory, which is analysed to determine phase fractions and the pathway followed over the course of the reaction^19^.

Previous studies^14,15^, particularly recent works^5,16^, have analysed selectivity in solid-state synthesis reactions based on first-principles thermodynamic data. Reference ^5^ suggested a thermodynamic threshold of 60 meV per atom above which the initial product formation can be reliably predicted. By contrast, for systems with multiple competing phases of comparable driving forces less than the threshold, the initial product was often determined by kinetics. For the Ba–Ti–O system, the formation energy difference between the product with the highest formation driving force (Ba_2_TiO_4_) and BaTiO_3_ and BaTi_2_O_5_ is ~51 meV per atom and 46 meV per atom, respectively. Hence, we expect synthesis outcomes in this system to be impacted by a temperature-dependent interplay between diffusive fluxes and reaction energies. Indeed, experimentally, the first observed product is generally Ba_2_TiO_4_ (ref. ^16^); however, products with a lower driving force are subsequently found, such as BaTi_2_O_5_ and BaTiO_3_. Using Ba–Ti–O as an exacting test case, we here present a general, predictive framework for solid-state synthesis outcomes that integrates rigorously computed ionic transport and first-principles thermodynamics with a cellular automata (ReactCA), a discrete computational model in which grid cells evolve based on local neighbour interactions (Fig. 3). As shown, predictions closely match four carefully characterized experiments across time and temperature.

## Kinetics in the Ba–Ti–O phase space

We consider two reactants (here BaO and TiO_2_) and nine possible solid-state reaction products in the Ba–Ti–O space (Fig. 2a, blue squares). We use BaO instead of BaCO_3_, as it is recognized that the experimental precursor BaCO_3_ decomposes to BaO at ~1,100 K (ref. ^17^), before any ternary reaction occurs^13^. We calculate the flux of constituent ions from the chemical potential difference across the interface and transport coefficients of Ba^2+^, Ti^4+^ and O^2−^ using Onsager analyses. These analyses are based on 5-ns molecular dynamics (MD) trajectories generated by machine learning interatomic potentials trained on ab initio molecular dynamics (AIMD) data for each liquid-like, non-crystalline analogue of nine possible products in the Ba–Ti–O system.

Using ionic fluxes of both Ba^2+^ and Ti^4+^, effective diffusion rate constants (*K*_D_) are estimated for each considered liquid-like interphase product (Methods). Figure 2b shows *K*_D_ for nine possible product compositions across the BaO/TiO_2_ interface for temperatures of 1,000–1,750 K. Interestingly, above 1,000 K, *K*_D_ in Ti-rich phases is more than an order of magnitude higher than that in the Ba-rich phases. In the Ti-rich phases, *K*_D_ increases by around an order of magnitude with every 250 K rise in temperature, and plateaus to a similar value for most phases at 1,750 K. On the other hand, *K*_D_ only increases by an order of magnitude in Ba-rich phases (Ba:Ti ratio > 1) on raising temperature by 750 K.

## Kinetics-informed cellular automaton simulations of Ba–Ti–O solid-state synthesis reactions

**Fig. 4:**
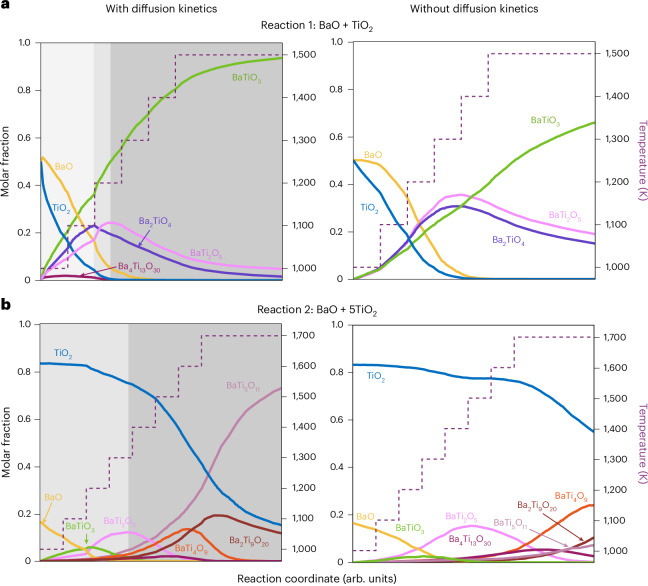
Reaction simulations using BaO and TiO_2_ precursors with different ratios using ReactCA, informed by both diffusive fluxes and reaction thermodynamics versus only thermodynamics. **a**,**b**, Reaction 1 (BaO + TiO_2_) (**a**) and reaction 2 (BaO + 5TiO_2_) (**b**). The shade of the grey background distinguishes the different regimes of reaction selectivity: the light grey region signifies the activation-controlled regime, the darker grey region signifies the kinetics-controlled regime and the dark region signifies the thermodynamics-controlled regime. Source data

**Fig. 5:**
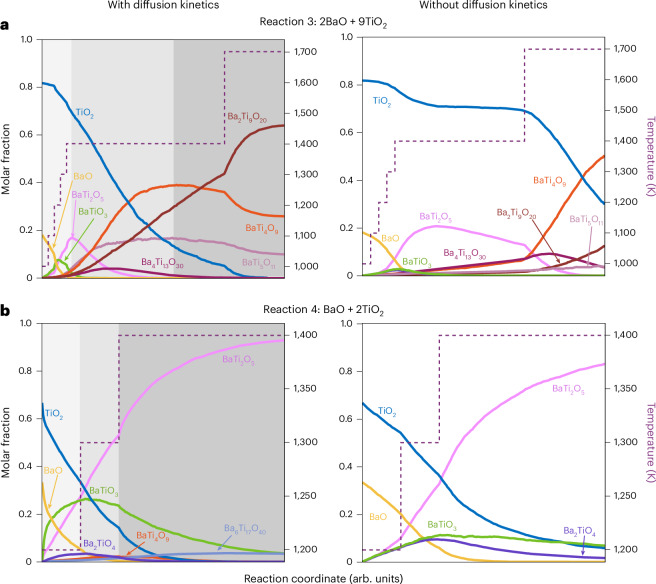
Reaction simulations using BaO and TiO_2_ precursors with different ratios using ReactCA, informed by both diffusive fluxes and reaction thermodynamics versus only thermodynamics. **a**,**b**, Reaction 3 (2BaO + 9TiO_2_) (**a**) and reaction 4 (BaO + 2TiO_2_) (**b**). The shade of grey background distinguishes the different regimes of reaction selectivity: the light grey region signifies the activation-controlled regime, where kinetics is slow for all compositions; the darker grey region signifies the kinetics-controlled regime, where kinetics dominates the phase selectivity among thermodynamically competitive phases; and the dark region signifies the thermodynamics-controlled regime, where kinetics is fast for all compositions. Source data

To compare temperature and time-dependent evolution of products with synthesis experiments reported elsewhere^13,15,16,18^, we use the recently developed cellular automaton simulation framework ReactCA^19^. ReactCA was designed to simulate solid-state reactions by modelling a three-dimensional grid of cells that evolve based on customizable local rules, incorporating both thermodynamic and kinetic inputs through a proxy for the reaction rate (a ‘scoring’ function). Here we extend the ReactCA framework by allowing the scoring function to depend on the instantaneous growth rate, which is a function of a calculated effective ionic diffusion constant of the liquid-like product phase (*K*_D_) at temperature *T*, a modified thermodynamic driving force ($\frac{\Delta {G}^{* }}{{k}_{{\rm{B}}}T}$) and a heuristic for Tammann’s rule^20^. Below the Tammann temperature, reaction rates are low but possible. Above it, rates increase with temperature due to both diffusion and thermodynamic contributions; however, at high temperatures, the saturation of diffusion rates shifts the balance in favour of thermodynamics-controlled outcomes. We simulate precursor Ba:Ti stoichiometries from 1:5 to 1:1 using the same heating profiles as the experiments (Figs. 4 and 5)^15,16,18,21^. Simulations using a scoring function that excludes the effective diffusion rates, that is, reaction rates based only on thermodynamics and Tamman’s rule are shown in the same figure for comparison.

Reaction 1 (Fig. 4a) represents the prototypical 1:1 BaO to TiO_2_ synthesis reaction, involving heating to 1,500 K followed by a brief annealing period^16,21^, resulting in BaTiO_3_ as the predominant product. In our simulation, the first products to form at low temperatures are Ba_2_TiO_4_ and BaTiO_3_. This agrees excellently with experimental results showing Ba_2_TiO_4_ as the primary impurity (up to 27 mol%)^13,16^ when the reaction was performed between 1,000 and 1,050 K. At higher temperatures, we find that Ba_2_TiO_4_ is consumed in favour of BaTi_2_O_5_, which emerges as the major impurity past 1,200 K, matching the data in refs. ^15,16^. With continued heating, BaTi_2_O_5_ is mostly consumed but persists as the primary impurity (<5 mol%). Indeed, in ref. ^21^, the reaction was found to be driven to completion when performed for >100 min at 1,300 K, giving almost phase-pure BaTiO_3_. Performing the same simulation without incorporating diffusion rates yields a qualitatively different outcome. Results from simulations that incorporate only the thermodynamics-based data as well as Tamman’s rule show a mixture of BaTiO_3_ (~70 mol%), Ba_2_TiO_4_ (~15 mol%) and BaTi_2_O_5_ (~20 mol%) after annealing at 1,500 K, with Ba_2_TiO_4_ and BaTi_2_O_5_ amounts peaking at similar temperatures. In particular, BaTiO_3_ accounts for only 70% of the product, in stark contrast to the nearly phase-pure BaTiO_3_ observed experimentally.

Reaction 2 (Fig. 4b) follows the experiments conducted earlier^22^ in which a 1:5 ratio of BaO and TiO_2_ was reacted at a series of temperatures (1,250–1,500 K). At low temperatures (<1,300 K), our simulation suggests small amounts of BaTiO_3_ (<10 mol%) and BaTi_2_O_5_ (~10 mol%) form initially and then convert to BaTi_5_O_11_, Ba_2_Ti_9_O_20_ and an intermediate BaTi_4_O_9_. BaTi_4_O_9_ reaches a maximum of ~15 mol% at 1,500 K before being consumed in favour of the high-temperature products BaTi_5_O_11_ and Ba_2_Ti_9_O_20_, together with the remaining precursor TiO_2_. Interestingly, only BaTi_5_O_11_, BaTi_4_O_9_ and unreacted rutile (TiO_2_) were present in the final product distributions of ref. ^22^. Since they do not specify the annealing or reaction times, we suggest that their experiment maps onto the early stages of our simulation, before substantial Ba_2_Ti_9_O_20_ formation. Continuing the reaction longer at a higher temperature would have promoted the formation of Ba_2_Ti_9_O_20_, which also qualitatively agrees with the experiments conducted in ref. ^23^ in which Ba_2_Ti_9_O_20_ forms at the expense of BaTi_4_O_9_ and TiO_2_ above 1,400 K.

In ref. ^18^, BaO and TiO_2_ in a 2:9 ratio (reaction 3) were reacted by rapidly increasing the temperature to ~1,400 K, followed by annealing for 3 h. The sample was then sintered at approximately 1,700 K for 6 h, yielding a Ba_2_Ti_9_O_20_ product with BaTi_5_O_11_ and BaTi_4_O_9_ impurities. In particular, Ba_2_Ti_9_O_20_ was predominantly formed at the expense of BaTi_5_O_11_ and BaTi_4_O_9_. In ref. ^18^, they also observe the relative rates of formation of the three phases as follows: BaTi_5_O_11_ > BaTi_4_O_9 _> Ba_2_Ti_9_O_20_. In our simulation (Fig. 5a), small amounts of BaTiO_3_ and BaTi_2_O_5_ initially form, and quickly convert to Ba_2_Ti_9_O_20_, BaTi_5_O_11_ and BaTi_4_O_9_. In particular, our results successfully reproduce the experimentally observed trend in the initial formation rates, following the sequence BaTi_5_O_11_ > BaTi_4_O_9_ > Ba_2_Ti_9_O_20_. Furthermore, and in qualitative agreement with experiments, the growth of BaTi_5_O_11_ and BaTi_4_O_9_ stagnates during the 1,400 K anneal and finally declines above 1,400 K, whereas Ba_2_Ti_9_O_20_ is continually produced at a positive rate. In ref. ^18^, trace quantities of Ba_4_Ti_13_O_30_ and some unreacted rutile (TiO_2_) were also detected when the reaction was performed below 1,400 K, which are also observed in our simulations.

Reference ^24^ provided a recipe to synthesize phase-pure BaTi_2_O_5_ by annealing a 1:2 BaO:TiO_2_ precursor ratio at three successive temperature steps: ~1,200 K, 1,300 K and 1,400 K, which we simulate through reaction 4 (Fig. 5b). After the first step, their sample primarily contained BaTiO_3_ and some amount of BaTi_2_O_5_. After the second annealing step, the BaTiO_3_ content was reduced and BaTi_2_O_5_ increased proportionately. After the third annealing step, close to phase-pure BaTi_2_O_5_ was observed, with BaTiO_3_ and Ba_6_Ti_17_O_40_ appearing as impurity phases. On longer heating time above 1,400 K, BaTiO_3_ and Ba_6_Ti_17_O_40_ (the equilibrium phases above 1,473 K) increase in phase fraction^24^. Our simulations show excellent agreement; we show a rapid formation of BaTiO_3_ as the majority phase in the first annealing step, which is overtaken by BaTi_2_O_5_ in the second annealing step and we recover phase-pure BaTi_2_O_5_ with trace (<5%) impurities of Ba_6_Ti_17_O_40_ and BaTiO_3_ as the final products.

Finally, in the study of the formation of BaTiO_3_ by the conventional solid-state reaction in air, ref. ^15^ mentions the formation of BaTi_4_O_9_ and Ba_4_Ti_13_O_30_ phases concurrently with BaTiO_3_ in the early stages of the reaction. They attribute the presence of these impurity phases to kinetic effects, that is, phases that—despite having only modest thermodynamic driving forces for formation—can nucleate and grow due to their rapid diffusion kinetics. Our simulations align well with experimental observations, showing the formation of these phases alongside BaTiO_3_, with their occurrence depending on the overall system composition. Ti-rich systems predominantly produce trace amounts of BaTi_4_O_9_ (reaction 4), whereas more compositionally balanced systems yield trace amounts of Ba_4_Ti_13_O_30_ (reaction 1).

## Discussion

By integrating rigorously computed ionic diffusion coefficients for a liquid-like reactive interphase into an effective diffusion rate constant (*K*_D_), our approach advances the a priori prediction of solid-state synthesis pathways and outcomes as a function of temperature and time. Figures 4 and 5 are in good agreement with carefully characterized experiments, accurately capturing both products and intermediates as functions of temperature and time. In particular, when performing the same simulations using only thermodynamic reactivity data and Tamman’s rule, the same framework often predicts the correct major product, whereas failing to accurately capture the intermediate products and major impurities, which are either absent or incorrectly identified.

**Fig. 6:**
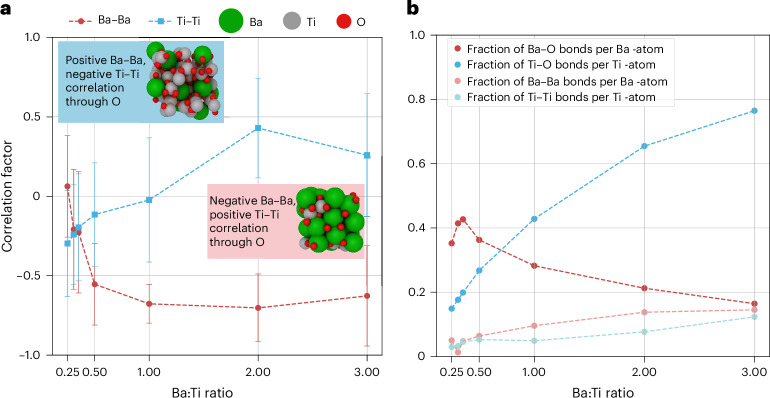
Analysis of correlation effects in liquid-like systems considered at 1,500 K. **a**, Correlation factors (ratio of distinct to self-transport coefficient) for Ba (shown in red) and Ti (shown in blue). Error bars are computed using the standard deviation across five MD trajectories. **b**, Local coordination environment analysis of liquid-like interphases, as a function of Ba:Ti ratio in the phase. On increasing the Ba content in the phase, a crossover point exists after which motifs of TiO_*x*_ clusters dominate with an increased Ba–Ba interaction through a bridging O (evidenced by the higher correlation factor and number of Ba–Ba bonds). Correspondingly, the distinct ion correlation of Ba–Ba becomes negative, implying repulsive behaviour between neighbouring Ba^2+^ ions, leading to a reduction in the mobility of Ba^2+^ ions. Source data

The strong agreement between experiments and ab initio predictions supports the hypothesis that transport through a liquid-like interphase matching the target phase approximates experimental conditions well. Using this insight, we analyse the impact of different liquid-like compositions on diffusion rates, which is expected to exhibit strong ion correlations^25,26^. Although such effects have been studied in detail in previous works on transport through concentrated ionic liquids^27,28^, an analysis of this nature has been absent from the discussion of solid-state reaction intermediates. To showcase the impact of correlated ionic motion, we plot the calculated distinct ion Onsager transport coefficients, normalized by the self-diffusion part of the correlation function for the cations (Ba^2+^ and Ti^4+^). Figure 6a depicts the normalized distinct Ba-ion and Ti-ion transport coefficients, compared with the fraction of Ba–O, Ti–O, Ba–Ba and Ti–Ti coordination per atom present in the liquid-like interphasial structures for each composition. The data reveal a strong trend between the degree of correlated motion between Ba^2+^ and Ti^4+^ and the local coordination environment. On increasing the Ba:Ti ratio in the system, the Ti^4+^ environment becomes dominated by O^2−^, which bridge other Ti^4+^, leading to higher fraction of TiO_*x*_ clusters. This, in turn, leads to an increase in the number of Ba–Ba coordination per Ba atom (Fig. 6b) and a higher degree of Ba–Ba correlated motion. The observation aligns with the computed distinct ion correlations: in Ba-rich phases, Ba^2+^ ions show negative correlations, indicating mutual repulsion under a chemical potential gradient, whereas Ti^4+^ ions exhibit smaller positive correlations, suggesting a weak tendency to cluster. We also note that the smaller size of Ti^4+^ and titanium’s known tendency towards network formation in glassy structures^29^, when compared with Ba^2+^, is consistent with the variation in transport on altering the Ba:Ti ratio of the system. Hence, the net Ba^2+^ movement slows compared with self-diffusion, reducing its mobility due to cross-correlated motion in Ba-rich interphases. A similar, but weaker, effect occurs in Ti-rich phases, causing the diffusion rate constant to peak at intermediate Ba:Ti ratios (Fig. 2b). To demonstrate how the correlated motion between the Ba and Ti ions affects the selective phase formation during synthesis, we compare the simulations with and without the cross-ion effects, that is, using only the self-diffusion estimates for ionic mobility (Supplementary Fig. 1). Although the results are qualitatively similar to the data in Figs. 4 and 5, there are key differences, primarily at the initial stage when reactions involve more Ba-rich phases. For example, reaction 1 does not form the experimentally verified Ba_4_Ti_13_O_30_ intermediate^15^. In reaction 3, adding cross-ion fluxes reduces the amount of initial Ba-rich intermediates, which agrees with earlier observations^18^. Furthermore, in reaction 3, only Ba_2_Ti_9_O_20_ and BaTi_4_O_9_ are experimentally observed in the final product distribution^18^, a trend that is more accurately captured in the simulation when cross-transport terms are included. When only the self-transport terms are considered, the simulation overestimates the amount of Ba_2_Ti_9_O_20_ and underestimates the BaTi_5_O_11_, and vice versa in reactions 2 and 3, respectively. We conclude that although self-diffusion terms capture most kinetic effects, incorporating cross-ion fluxes is crucial to accurately capture the relative phase formation. These cross-effects are expected to be even more substantial in reactions like ion exchange or metathesis, involving multiple anion groups^30,31^.

Furthermore, by analysing the difference between the simulations based on thermodynamic data only and thermodynamics combined with kinetic information, we can identify three temperature regimes for selectivity in the Ba–Ti–O system: activation-controlled, kinetics-controlled and thermodynamics-controlled regimes. At low temperatures (below ~1,100 K for the Ba–Ti–O system), where diffusion kinetics is slow across all products, phases that align reasonably with the precursor ratio and which exhibit substantial reaction energies Δ*G*_rxn_ are favoured (activation-controlled regime). At intermediate temperatures (1,100 K < *T* < 1,700 K), diffusion kinetics favour the formation of certain compositions among the possible phases (kinetics-controlled regime). At high temperatures (*T* > 1,700 K), close to the melting point of the system, kinetics is fast for all ionic species, and the mixture of phases closest to the globally set composition on the thermodynamic hull form to establish thermodynamic equilibrium. This insight agrees with existing theories of solid-state reactivity^1,32–34^. For example, BaTiO_3_ and BaTi_2_O_5_, which exhibit large negative formation energies on the Ti-rich side of the hull and relatively fast kinetics (Fig. 2), tend to form at lower temperatures (reactions 2 and 3). Raising the temperature above 1,700 K, corresponding to the thermodynamic regime, promotes the formation of BaTi_5_O_11_ and Ba_2_Ti_9_O_20_, which is thermodynamically the most favourable product mixture for the given precursor ratio.

Finally, we assess the impact of structural similarity in solid-state synthesis. Several prior works^5,18,35^ argue that structural similarity or ‘templating’ between the precursors, intermediates and products promote the formation of specific phases. Indeed, in ref. ^18^, it was hypothesized that Ba_2_Ti_9_O_20_ forms above 1,373 K only when the intermediate BaTi_5_O_11_ provides a lowering of interfacial and strain energies by the templating of the Ba_2_Ti_9_O_20_ phase onto BaTi_5_O_11_. Here we reproduce the product distribution of ref. ^18^ without incorporating any structural similarity effects. Specifically, we find similar relative rates of formation for the three ternary phases in the final product distribution (BaTi_5_O_11_ > BaTi_4_O_9_ > Ba_2_Ti_9_O_20_) during the early stages of reactions 2 and 3 (Figs. 4 and 5) and that, importantly, Ba_2_Ti_9_O_20_ is only thermodynamically stable above 1,373 K, as indicated by the BaO–TiO_2_ phase diagram^36^. The modest driving force for its formation (Fig. 2) accounts for its appearance only at temperatures above 1,373 K in both experiments in ref. ^18^ and our simulations, as well as its absence in the shorter reaction times used by others^16^. To generally analyse the structural effects in the Ba–Ti–O system, we included the nucleation rate computed using the approach in ref. ^35^ Their metric shows that BaTiO_3_ and BaTi_2_O_5_ exhibit the lowest nucleation barriers and should dominate at low to moderate temperatures (Supplementary Table 1). However, Ba_2_TiO_4_ often forms as a low-temperature intermediate in both experiments and simulations, which cannot be explained by this approach. Hence, although structural similarity serves as a valuable conceptual framework for synthesis reactions that proceed topochemically, we find that the proposed approach better explains product outcomes when the reactants undergo substantial structural transformation and/or decompose entirely. We expect our method to be well suited to study this thermodynamics–kinetics interplay in more diffusion-limited solid-state synthesis reactions, with the ability to perform such simulations in high throughput to search for better recipes of both existing and unrealized materials that are possibly kinetically prevented from forming.

## Methods

To capture ion dynamics effects in dense liquid-like or non-crystalline phases, we adapt the framework of ref. ^38^ to obtain both self- and correlated diffusive transport coefficients in the form of the Onsager transport matrix. Robust estimates of transport coefficients are obtained from nanosecond-long MD trajectories, using an atomic cluster expansion (ACE)-based machine learning interatomic potentials^39^, trained on 75 AIMD trajectories from non-crystalline systems. The resulting kinetic data, as well as thermodynamic data from the MP, are implemented in the ReactCA simulation framework^19^, which allows for describing both spatial and temporal phase evolution over the course of a prescribed solid-state reaction. Below, we describe the components of the simulation framework in more detail.

### Accelerating AIMD using ACE

We use the MPMorph^40,41^ workflow, as implemented in the atomate2 Python package, to generate amorphous configurations for training ACE. Sample random amorphous structures of the desired composition are first generated using packmol. The MPMorph workflow begins by adjusting the volume of this structure to 0.8 and 1.2 times the initial volume, followed by a 4-ps *NVT* AIMD run to determine the equation of state at the given temperature. A trial volume is then selected based on the equation of state for another 4-ps *NVT* AIMD run to check for energy convergence. If the energy converges, a 20-ps AIMD ‘production’ run is performed to equilibrate the structure at this volume. If not, the workflow continues to iteratively rescale the volume until energy convergence is achieved. Once a converged volume is found, it is used for the 20-ps production run. The workflow is run on high-performance computing resources using the jobflow and fireworks Python packages.

Compositions according to BaTiO_3_, Ba_2_TiO_4_, BaTi_2_O_5_ and BaTi_5_O_11_ were run at 1,000, 1,250 and 1,500 K each, and frames were sampled every 100 fs to ensure sufficient variety in the sampled configurations. The sampled frames were used in addition to MP data in the Ba–Ti–O chemical space to train ACE potentials using the pacemaker^42,43^ Python package. The ACE potential is parameterized with 500 basis functions per element, considering neighbourhood lists within 5 Å. Following ref. ^44^, a higher-order Finnis–Sinclair embedding function is used. Training is performed by hierarchically growing the potential using a power-order based ‘ladder-fitting’ approach^43^ with a higher weight on the loss contribution from forces (99%) for 2,000 iterations. Once convergence of the training was obtained, the potential was fine-tuned by reducing the force loss weight to 90% for another 2,000 iterations and keeping the shape and complexity of the potential fixed.

Active learning was performed using the extrapolation grade and high-temperature MD approach described in ref. ^45^. We generate randomly packed structures using packmol for compositions corresponding to BaTi_5_O_11_, Ba_2_Ti_9_O_20_, BaTi_4_O_9_, Ba_4_Ti_13_O_30_,Ba_6_Ti_27_O_40_, BaTi_2_O_2_, BaTiO_3_, Ba_2_TiO_4_ and Ba_3_TiO_5_ as input for ACE-based *NVT* MD simulations for 100 ps (100,000 steps) at 2,000 K. We use the extrapolation grade, which quantifies the deviation of the test configurations sampled through ACE-MD from those encountered in the training data to filter out structures that are sufficiently different from the training data. During the high-temperature MD simulations, all configurations encountered by the model with an extrapolation grade of more than 5 are considered for active learning, and the MD simulation is terminated if the model encounters a configuration with extrapolation grade exceeding 100. A subset of the collected structures is determined using the D-optimality criterion^45^ to ensure diversity in the active learning data, and DFT single-point calculations are performed to compute the DFT energy and forces. The trained potential is then retrained with the active learning data for an additional 1,000 iterations, and this active learning loop is repeated four times. In total, including the data acquired during active learning, the training dataset consisted of 4,628 structures containing 492,216 atoms.

It is important to highlight that the objective here is not to develop a potential capable of addressing a broad variety of tasks (such as crystal relaxation, property calculations and so on) within the Ba–Ti–O chemical space, as has been successfully achieved with simpler systems. Specifically, the goal of the trained potential is to obtain longer-time *NVT* MD trajectories with similar-to-DFT accuracy on the amorphous liquid-like configurations. With this purpose in mind, benchmarks for the quality of the trained model are discussed in Supplementary Section 3.

### Onsager transport framework for a solid-state reaction interface

In the framework of linear irreversible thermodynamics, the diffusive flux induced in an ion *i* due to a driving force can be written as^27,38^

1 $${J}_{i}=-\mathop{\sum }\limits_{j}{L}_{ij}\nabla {\widetilde{\mu }}_{j}.$$

This expression considers the effect of driving forces ($\{\nabla {\widetilde{\mu }}_{j}\}$) on all ions present in the system to the flux experienced by ion *i* through the Onsager transport matrix (**L** = [*L*_*ij*_]). The transport coefficient *L*_*ij*_ is then computed from an MD trajectory using the differential form of the Green–Kubo relations^27,38^:

2 $${L}_{ij}=\frac{1}{6{k}_{{\rm{B}}}TV}\mathop{{\lim}}\limits_{t\to \infty }\frac{\mathrm{d}}{{\mathrm{d}}t} < \mathop{\sum }\limits_{\alpha }\left[{{\bf{r}}}_{\alpha }^{{\rm{i}}}{(\rm{t})}-{{\bf{r}}}_{\alpha }^{{\rm{i}}}(0)\right]\cdot \mathop{\sum }\limits_{\beta }\left[{{\bf{r}}}_{\beta }^{{\rm{j}}}(\rm{t})-{{\bf{r}}}_{\beta }^{\,{\rm{j}}}(0)\right] > .$$

Here *V* and *T* are the volume and temperature of the system, respectively; ${{\bf{r}}}_{\alpha }^{i}(t)-{{\bf{r}}}_{\alpha }^{i}(0)$ is the displacement of the *α*th particle of species *i* at time *t*. The self-transport coefficient, ${L}_{ii}^{{\rm{self}}}$, can be computed using a similar relation:

3 $${L}_{ii}^{\mathrm{self}}=\frac{1}{6{k}_{{\rm{B}}}TV}\mathop{{\mathrm{lim}}}\limits_{t\to \infty }\frac{{\rm{d}}}{{\rm{d}}t} < \mathop{\sum }\limits_{\alpha }{\left[{{\bf{r}}}_{{\rm{\alpha }}}^{{\rm{i}}}({\rm{t}})-{{\bf{r}}}_{{\rm{\alpha }}}^{{\rm{i}}}(0)\right]}^{2} > .$$

The self-transport coefficient of an ion *i* is related to the Nernst–Einstein estimate of diffusivity, also called the self-diffusion coefficient (*D*_*i*_) through the following relation:

4 $${L}_{ii}^{{\rm{self}}}=\frac{{{D}}_{i}{c}_{i}}{{k}_{{\rm{B}}}T},$$

where *c*_*i*_ is the concentration of species *i*. This self-term exactly describes ionic mobility for dilute systems, where diffusion is ideal. Practically, *L*_*ij*_ is the slope of a linear fit (the diffusive regime) of the time correlation function, $< {\Sigma }_{\alpha }[{{\bf{r}}}_{\alpha }^{i}(t)-{{\bf{r}}}_{\alpha }^{i}(0)]\cdot {\Sigma }_{\beta }[{{\bf{r}}}_{\beta }^{j}(t)-{{\bf{r}}}_{\beta }^{j}(0)] >$, with time *t*. Hence, a linear regime of at least 200 ps (200,000 steps) is used in the time correlation function versus time plot to compute all the transport coefficients. More details on how the transport coefficients are fit, as well as other caveats when using this theory on solid-state reactions, are provided in Supplementary Section 4.

### Deviations in diffusion due to correlated ion movement

The deviation in effective diffusion coefficients can be studied through the cross-ion correlations, which are quantified by the off-diagonal terms of **L**, and the distinct ion correlations, which are computed as

5 $${L}_{ii}^{{\rm{distinct}}}={L}_{ii} - {L}_{ii}^{{\rm{self}}}.$$

In this work, we focused on the effect of correlations amongst the cation pairs Ba–Ba and Ti–Ti through the corresponding distinct ion correlation terms. To compare the correlation terms between phases, we calculate the distinct correlation normalized by the self-part of the correlation function, which we call the correlation factor (*f*):

$${f}_{i}=\frac{{L}_{ii}^{{\rm{distinct}}}}{{L}_{ii}^{{\rm{self}}}}=\frac{{L}_{ii}}{{L}_{ii}^{{\rm{self}}}}-1.$$

The correlation factor typically exhibits values between –1 and +1, with values close to 0 implying uncorrelated motion between ions of type *i*. Additionally, we analyse the local coordination environment within the liquid-like phases by counting all geometrically feasible bonds for each ion within a cut-off radius of 5 Å. This approach captures spatial correlations or bonding interactions within both first and second coordination shells. Both shells are included because cations typically interact through bridging O ions. This enumeration is performed using the CrystalNN algorithm implemented in pymatgen^46,47^.

### Estimating reaction kinetics from atomistic transport

The liquid-like structures generated when training the ACE potential serve as inputs for *NVT* MD simulations using the ACE potential. These simulations are conducted for 5 ns with a 1-fs time step at temperatures of 750, 1,000, 1,250, 1,500 and 1,750 K, using the Langevin thermostat with a friction factor of 0.01 fs^−1^. To obtain better statistics on the computed transport coefficients, five frames from the production run of the ACE-based MPMorph flow are used as starting configurations for the *NVT* MD runs, leading to five replicate MD runs for each composition.

Following ref. ^48^, as the first order, we approximate the driving forces $\nabla {\widetilde{\mu }}_{j}$ in equation (1), which are electrochemical potential gradients, by the bulk chemical potential gradient for species *j*, ∇*μ*_*j*_. These gradients are obtained through the relevant chemical potential diagram for the chemical system, which is built using pymatgen^47^ with data for all crystalline phases from the MP (Supplementary Fig. 5). We approximate the gradient in chemical potential for species *j* between the precursors (or in general, the reactants) of a solid-state reaction by the shortest distance on chemical potential diagram between domains corresponding to the precursors (reactants), say *α* and *β*, divided by the thickness of the product layer (*h* in Supplementary Fig. 8):

6 $$\nabla {\widetilde{\mu }}_{j}\approx \nabla {\mu }_{j}=\frac{\min \left(\,{\mu }_{j}^{\alpha }-{\mu }_{j}^{\beta }\right)}{h}.$$

This approach ensures that when the phases are in thermodynamic equilibrium, the reaction driving force and the resulting flux are zero^30^. The effective ‘diffusion rate constant’ (*K*_D_) is defined as follows:

7 $${K}_{{\rm{D}}}=\mathop{\sum }\limits_{i}\mathop{\sum }\limits_{j}\frac{\left|{L}_{ij}^{\gamma }{V}^{\,\gamma }\times \min \left(\,{\mu }_{j}^{\alpha }-{\mu }_{j}^{\,\beta }\right)\right|}{{n}_{i}^{\gamma }{N}_{{\rm{A}}}}.$$

where *V*^*γ*^ is the molar volume of the product *γ*, ${n}_{i}^{\gamma }$ is the fraction of atoms of species *i* in *γ* and *N*_A_ is the Avogadro number. We assume a core–shell model for the formation of the product (the derivation is shown in Supplementary Section 6), with the growth of *γ* supported by the transport of Ba^2+^ and Ti^4+^ across the interface. In particular, the growth rate varies with the thickness of the product phase, and decreases as the reaction proceeds. For a purely diffusion-limited case for the geometry shown in Supplementary Fig. 8, the rate equation can be solved to give^49^

8 $$1-{(1-y)}^{1/3}=\frac{\sqrt{2{K}_{{\rm{D}}}t}}{{r}_{0}},$$

where *y* (1 ≥ *y* ≥ 0) is the degree of completion of the reaction and *r*_0_ is the radius of the precursor powder particle, reminiscent of the Jander equation, which is commonly used to fit kinetic models to the solid-state reaction data^6,7^. In this study, we assume that powder particles are well mixed and uniform in size, allowing us to analyse the kinetic feasibility of a solid-state reaction using the term $\frac{\sqrt{{K}_{{\rm{D}}}}}{{r}_{0}}$, where ${r}_{{\rm{B}}{\rm{a}}{\rm{O}}}={r}_{{\rm{T}}{\rm{i}}{{\rm{O}}}_{2}}={r}_{0}$. The parameter *r*_0_ is set uniformly for all reactions in our simulations. Specifically, we assign *r*_0_ a value of 10^−1^ µm, which is representative of particle sizes commonly used in solid-state synthesis.

We emphasize that this formulation is not applicable for all solid-state reactions, as the ${\widetilde{\mu }}_{j}\approx {\mu }_{j}$ approximation does not hold when species exhibit variable oxidation states, or there exists a substantial amount of charge transfer between species, both of which are known to occur in many solid-state reactions involving transition metals. In the present study, all phases considered have species in only a single oxidation state: +2 for Ba, +4 for Ti and –2 for O. Furthermore, the phenomenological expression for the flux (equation (1)) is defined under the centre-of-mass frame of reference, which imposes constraints on the total number of independent material fluxes in the system for incompressible systems. These constraints are described in Supplementary Section 4.

### Simulation of solid-state synthesis reactions with ReactCA

A cellular automaton framework was recently developed (ReactCA)^19^ to simulate the evolution of mixtures of powders over the course of solid-state reactions. The reaction vessel is discretized into voxels (cells) and each voxel represents a powder particle of a given phase. At a given temperature, all possible reactions that can occur between any other powders (phases) in the system are enumerated combinatorially with an approach developed as part of a previous work^3^, and are scored using a scoring function. The system is initialized with a random distribution of two phases (in this case, representing two well-mixed precursor powders of uniform size). At every step of the simulation, two reactants are selected, and a reaction is probabilistically selected from the enumerated reactions based on their scores. Then, the reactants are probabilistically replaced with products based on the stoichiometry of the reaction. This process is repeated millions of times throughout the simulation to accurately model the evolution of phases within the reaction vessel. In the original publication^19^, reaction scores (that is, rates) between neighbouring particles were estimated based on the reaction thermodynamics (as estimated by zero-temperature formation energies from the MP in conjunction with a machine learning estimate of the vibrational Gibbs free energy^37^) and machine learning estimates of the melting point of the precursor materials^50^ (a preliminary proxy for the kinetic facility of the reaction). In this work, given a reaction *α* + *β* → *γ*, we incorporate the diffusive fluxes using a new scoring function *S* for ReactCA, as follows.

9 $$\begin{array}{l}\begin{array}{l}S={\sigma }_{1}\left(\frac{{K}_{\mathrm{D}}}{{r}_{0}^{2}s}\times \frac{\Delta {G}^{\ast }}{{k}_{\mathrm{B}}T}\right)\times {\sigma }_{2}\left(\frac{T}{{T}_{{\rm{m}},\mathrm{reactant}}}\right)\\ {\sigma }_{1}(x)=\frac{1}{3}\log [1+\exp (ax)]\\ {\sigma }_{2}(x)=\frac{1}{2}\log [1+\exp (bx-c)]\\ \Delta {G}^{\ast }=1+\mathrm{erf}(-d(\Delta {G}_{{\mathrm{rxn}}}+e))\\ {K}_{\mathrm{D}}=\mathop{\sum }\limits_{i}\mathop{\sum }\limits_{j}\frac{\left|{L}_{ij}^{\gamma }{V}^{\,\gamma }\times {\mathrm{min}}\left(\,{\mu }_{j}^{\alpha }-{\mu }_{j}^{\,\beta }\right)\right|}{{n}_{i}^{\gamma }{N}_{\mathrm{A}}}\end{array}\end{array}$$

The form of this scoring function is an extension to the scorer present in ReactCA^19^, using the same constants *a*, *b*, *c*, *d*, *e*. The factor *s* is set equal for all reactions considered, to appropriately scale the scores to be stable in the CA simulation and to ensure the reaction between the precursors initiate at temperatures above the decomposition temperature of BaCO_3_ (*s* = 5 × 10^13^). This scoring function has a ‘soft’ activation towards Tammann’s rule^20^, implemented through the *σ*_2_ part of the function, and a ‘switching-on’ behaviour towards reactions with negative reaction free energies (Δ*G*_rxn_) through the error function. For more details on these aspects of the scoring function, we refer to ref. ^19^. In this work, we implement explicit, quantitative transport diffusion constants in the liquid-like product phase (*K*_D_) through a soft activation with the *σ*_1_ function, which is also a soft-plus function. This function also takes the modified Δ*G*_rxn_ and the temperature into account. Hence, below the Tammann temperature ($\frac{T}{{T}_{{\rm{m}},{\rm{r}}{\rm{e}}{\rm{a}}{\rm{c}}{\rm{t}}{\rm{a}}{\rm{n}}{\rm{t}}}}\le 0.67$), the scores are non-zero, but only slightly positive. Above the Tammann temperature, reactions with a slightly positive Δ*G*_rxn_ or with low ionic fluxes have a non-zero but small score assigned to them. With increasing reaction temperature, the *σ*_2_ part of the function increases, thereby elevating the reaction rate. Similarly, raising the temperature also increases the diffusion rate constant *K*_D_ (often in a superlinear fashion), which generally boosts the output of the *σ*_1_ part of the scoring function. However, as demonstrated in this work, *K*_D_ approaches a saturation point with increasing temperature, which, in turn, causes the output of *σ*_1_ to also level off or even decrease at very high temperatures. This effect leads to the thermodynamic regime of phase selectivity when the reaction temperatures are close to or above the melting point. Finally, the scoring function maintains backward compatibility with the old scoring function, which is particularly important when kinetic transport parameters are not available for some or all phases in the system.

### DFT calculations

DFT single-point calculations and AIMD calculations were performed using the Vienna ab initio simulation package^51,52^ and the Perdew–Burke–Ernzerhof^53^ formulation of generalized gradient approximation with projector augmented-wave potentials^54,55^. Furthermore, to minimize computation requirements, all AIMD calculations were performed using the Γ point only and were non-spin polarized. For more details on the parameters used, we refer to the MPMorphMDSet class in atomate2. To construct the high-temperature phase diagrams, a machine learning estimator for the vibrational contribution to entropy^37^ was used to estimate the finite-temperature vibrational Gibbs free energies for all phases.

## Online content

Any methods, additional references, Nature Portfolio reporting summaries, source data, extended data, supplementary information, acknowledgements, peer review information; details of author contributions and competing interests; and statements of data and code availability are available at 10.1038/s41563-026-02596-5.

## Supplementary information

Supplementary Information Supplementary Figs. 1–11, Tables 1–3 and Sections 1–8.

Supplementary Data 1 Fitted transport coefficients for all phases considered at temperatures from 750–1,750 K (Supplementary Figs. 8 and 9).

## Source data

Source Data Fig. 2 Energies of all Ba–Ti–O phases from the MP and convex hull data at 600 °C (Fig. 2a); effective diffusion rate constants at temperatures from 1,000 to 1,750 K in all phases considered in this study (Fig. 2b).

Source Data Fig. 4 Phase traces (molar fraction versus reaction step) and temperature profiles for all phases appearing in ReactCA simulations of reactions (Fig. 4a,b).

Source Data Fig. 5 Phase traces (molar fraction versus reaction step) and temperature profiles for all phases appearing in ReactCA simulations of reactions (Fig. 5a,b).

Source Data Fig. 6 Correlation factor of Ba–Ba and Ti–Ti in the phases considered in this study (Fig. 6a); coordination environment indicators (coordination numbers) of Ba–O, Ba–Ba, Ba–Ti and Ti–O in the phases considered in this study (Fig. 6b).

## Data Availability

The data supporting the findings of this study are available within the article and its Supplementary Information. DFT and MD data (first and last trajectory frames) generated as part of this study are provided in Supplementary Information and also available via figshare at 10.6084/m9.figshare.28207292.v3 (ref. ^56^). Source data are provided with this paper.

## Code availability

Code used to generate and run the computational workflows from this study are a part of the atomate2 (https://github.com/materialsproject/atomate2) and rxn-ca (https://github.com/mcgalcode/rxn-ca) packages. Additional code necessary for the analysis done in this study is provided in Supplementary Information, and also available via figshare 10.6084/m9.figshare.28207292.v3 (ref. ^56^) and GitHub (https://github.com/vir-k01/py-OATS).
