# Supplementary material for: Ion correlations explain kinetic selectivity in diffusion-limited solid-state synthesis reactions
Source: Nat Mater. 2026 Apr 28;25(8):1385–92. doi: 10.1038/s41563-026-02596-5 (PMC13421335; doi:10.1038/s41563-026-02596-5)
Supplement: Supplementary file 1 — Supplementary Figs. 1–11, Tables 1–3 and Sections 1–8. [file 41563_2026_2596_MOESM1_ESM.pdf]

nature materials

Supplementary information

https://doi.org/10.1038/s41563-026-02596-5

Ion correlations explain kinetic selectivity in diffusion-limited solid-state synthesis reactions

In the format provided by the authors and unedited

Supplementary Information

Contents

S1 ReactCA simulations without cross-ion transport effects **S2**

S2 Nucleation barrier estimation using PIRO **S3**

S3 Validation of trained Atomic Cluster Expansion Potential **S4**

S3.1 Training and Test Loss S4
S3.2 Binary elemental interactions S6
S3.3 Equation of State test S6
S3.4 RDFs and short range order S7

S4 Adapting Onsager transport framework to diffusive transport in amorphous solids **S8**

S5 Chemical Potential Diagram for Ba-Ti-O system **S10**

S6 Derivation of rate across a spherical powder interface **S10**

S7 Computed Onsager transport coefficients **S12**

S8 ReactCA simulation step and scoring parameters **S13**

S1 ReactCA simulations without cross-ion transport effects

Reactions 1-4, were simulated using only the "self" transport fluxes in the scoring function. The scoring function for this case is given by:

$$S = \sigma_1(\frac{K_D}{r_0^2 s} \times \frac{\Delta G^*}{k_B T}) \times \sigma_2(\frac{T}{T_{m,reactant}})$$
$$K_D = \Sigma_j \frac{|L_{jj}^{\text{self},\gamma} V^\gamma \times \min(\mu_j^\alpha - \mu_j^\beta)|}{n_j^\gamma N_A} \tag{S2}$$

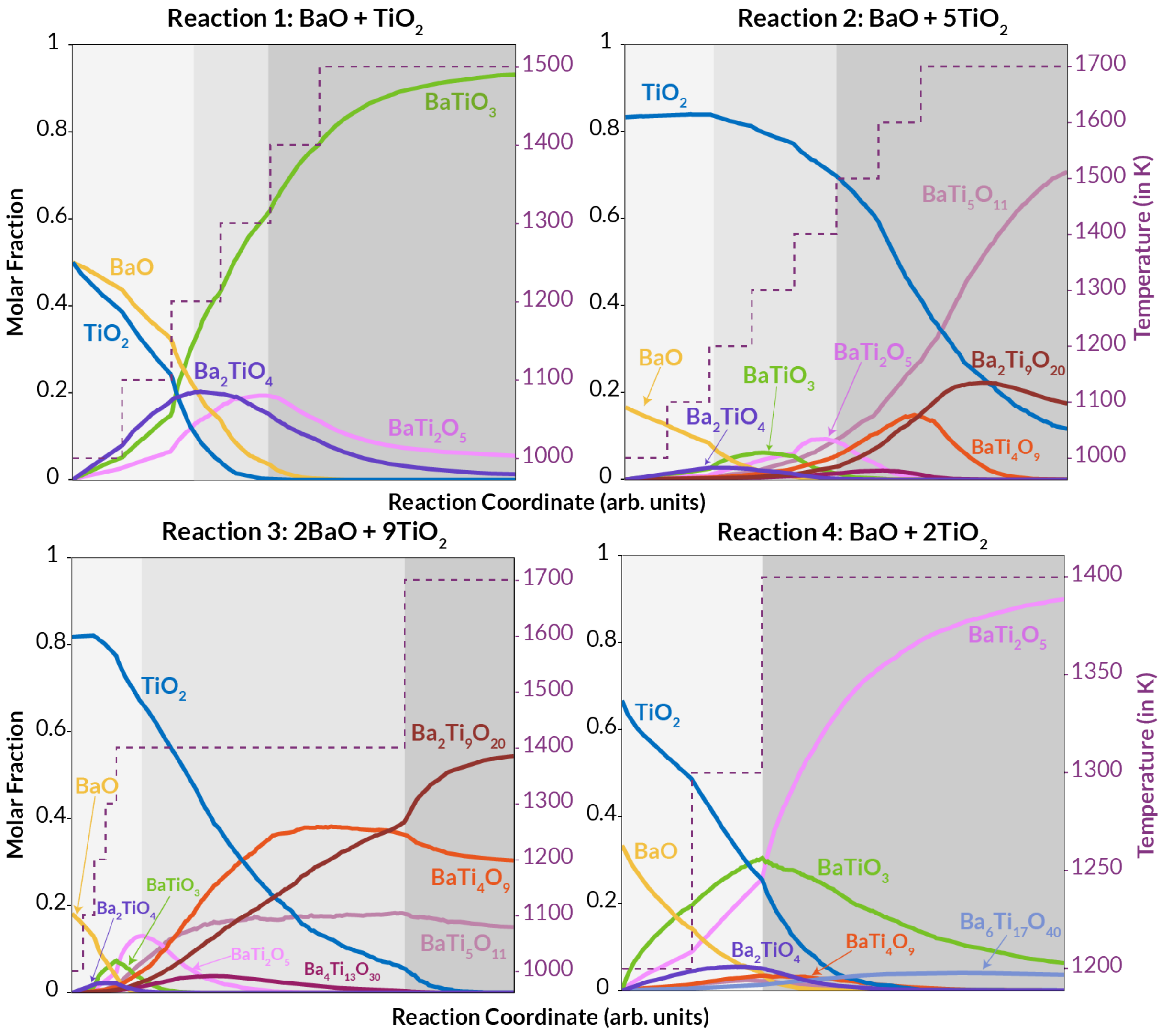

Figure S1: ReactCA simulation incorporating only self-diffusion based transport fluxes into the scoring function.

Here, L_{jj}^{self} can be estimated from the self-diffusion coefficient of specie j (D_j) and the concentration

of j (c_j) in the product layer at temperature T as:

$$L_{jj}^{\text{self}} = \frac{D_j c_j}{k_B T} \tag{S3}$$

Figure S1 shows the results of the simulations. The final product distributions are qualitatively very similar to the simulations from the manuscript which utilized the full transport matrix with cross-effects in the scoring function.

S2 Nucleation barrier estimation using PIRO

In order to consider nucleation kinetics into our score, we also experimented with the approach of using lattice matching/structural similarity between precursors and solid-state products as a proxy for the degree of heterogeneous nucleation as described in the PIRO approach. Table S1 shows the estimated nucleation barrier (ΔG^*) and rate of nucleation ($J_{\text{nuc}} \equiv \exp \frac{-\Delta G^*}{k_B T}$) for the formation of the products considered in this study from three precursor combinations: BaO + TiO_2, BaO_2 + TiO_2, $BaCO_3$ + TiO_2. Such barriers do not explain the formation of Ba_2TiO_4 in the early stages of the reaction, which has been experimentally observed to occur.

**Reaction**	$\Delta G*$	$J_{\mathbf{nuc}}$
$BaO + TiO_2 \longrightarrow BaTiO_3$	0.0435	**0.713977**
$BaO + TiO_2 \longrightarrow Ba_2TiO_4$	1.0888	**0.0002**
$BaO + TiO_2 \longrightarrow Ba_6Ti_{17}O_{40}$	6.2668	0.0
$BaO + TiO_2 \longrightarrow Ba_4Ti_{13}O_{30}$	6.7435	0.0
$BaO + TiO_2 \longrightarrow Ba_2Ti_9O_{20}$	8.7711	0.0
$BaO + TiO_2 \longrightarrow BaTi_2O_5$	0.3663	**0.0588**
$BaO + TiO_2 \longrightarrow Ba_3TiO_5$	1.8074	0.0
$BaO + TiO_2 \longrightarrow BaTi_4O_9$	1.5753	0.0
$BaO + TiO_2 \longrightarrow BaTi_5O_{11}$	5.8769	0.0
$BaO + TiO_2 \longrightarrow BaTi_6O_{13}$	2.3497	0.0
$BaO_2 + TiO_2 \longrightarrow BaTiO_3 + O_2$	0.5409	0.0152
$BaO_2 + TiO_2 \longrightarrow Ba_2TiO_4 + O_2$	99.9718	0.0
$BaO_2 + TiO_2 \longrightarrow Ba_6Ti_{17}O_{40} + O_2$	39.3189	0.0
$BaO_2 + TiO_2 \longrightarrow Ba_4Ti_{13}O_{30} + O_2$	34.8828	0.0
$BaO_2 + TiO_2 \longrightarrow Ba_2Ti_9O_{20} + O_2$	48.8114	0.0
$BaO_2 + TiO_2 \longrightarrow BaTi_2O_5 + O_2$	2.1875	0.0
$BaO_2 + TiO_2 \longrightarrow Ba_3TiO_5 + O_2$	26.5411	0.0
$BaO_2 + TiO_2 \longrightarrow BaTi_4O_9 + O_2$	9.6281	0.0
$BaO_2 + TiO_2 \longrightarrow BaTi_5O_{11} + O_2$	33.4494	0.0
$BaO_2 + TiO_2 \longrightarrow BaTi_6O_{13} + O_2$	16.2007	0.0
$BaCO_3 + TiO_2 \longrightarrow BaTiO_3 + CO_2$	0.1441	0.328
$BaCO_3 + TiO_2 \longrightarrow Ba_2TiO_4 + CO_2$	1.7433	0.0
$BaCO_3 + TiO_2 \longrightarrow Ba_6Ti_{17}O_{40} + CO_2$	28.4083	0.0
$BaCO_3 + TiO_2 \longrightarrow Ba_4Ti_{13}O_{30} + CO_2$	44.2047	0.0
$BaCO_3 + TiO_2 \longrightarrow Ba_2Ti_9O_{20} + CO_2$	39.2851	0.0
$BaCO_3 + TiO_2 \longrightarrow BaTi_2O_5 + CO_2$	2.3927	0.0
$BaCO_3 + TiO_2 \longrightarrow Ba_3TiO_5 + CO_2$	0.8953	0.001
$BaCO_3 + TiO_2 \longrightarrow BaTi_4O_9 + CO_2$	6.1428	0.0
$BaCO_3 + TiO_2 \longrightarrow BaTi_5O_{11} + CO_2$	23.7557	0.0
$BaCO_3 + TiO_2 \longrightarrow BaTi_6O_{13} + CO_2$	6.1576	0.0

Table S1: ΔG^* values, and J_{nuc} computed at 1500K using the PIRO approach.

S3 Validation of trained Atomic Cluster Expansion Potential

We reiterate that the purpose of the trained potential is to perform high-temperature, long time-scale molecular dynamics of amorphous configurations in the Ba-Ti-O chemical space. As such, we here demonstrate that the trained potential is accurate for this particular task.

S3.1 Training and Test Loss

The RMSE of energies and forces of the trained potential for the Ba-Ti-O system are given in Table S2. A parity plot comparing the predictions for energies and norm of forces of the potential to the ground truth data is shown in Figure S2.

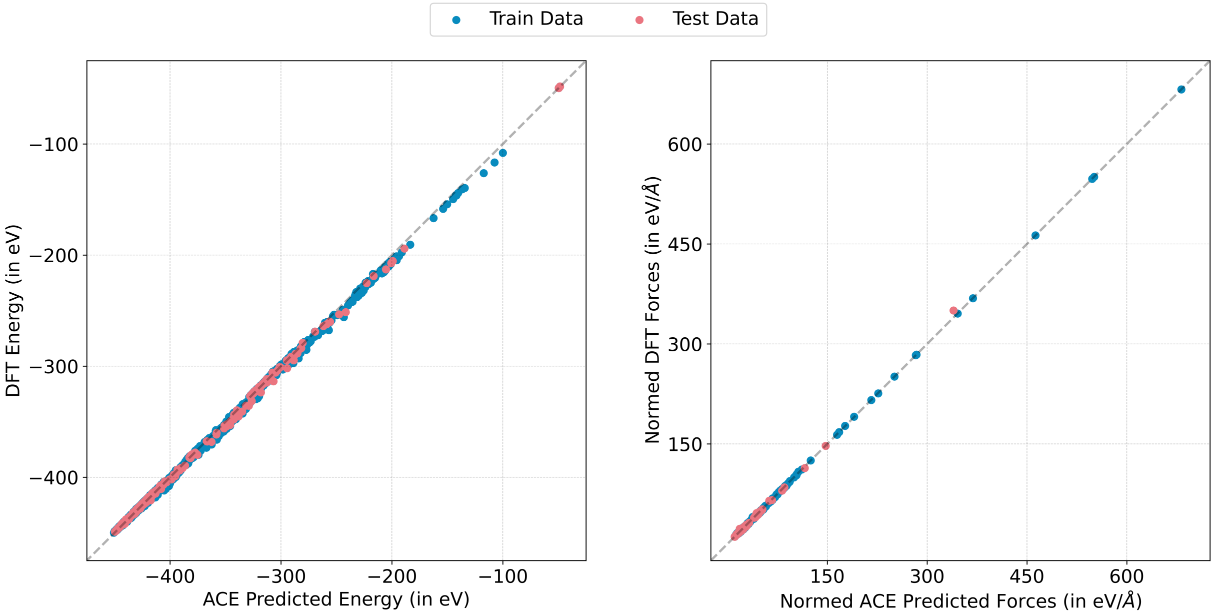

Figure S2: Parity plot of predicted (ACE) and ground truth for Ba-Ti-O system (DFT), A: energies, B: norm of forces.

Metric	Energy (in meV/atom)	Force (in meV/A)
RMSE	16.50	239.20
MAE	10.90	168.31

Table S2: Metrics to quantify performance of the trained ACE potential w.r.t ground truth DFT data. RMSE: Root Mean Squared Error, MAE: Mean Absolute Error.

S3.2 Binary elemental interactions

A quick way to assess the quality of an interatomic potential is to verify whether it accurately captures the equilibrium bond lengths, bond energies, and core repulsive behavior observed in physical bonds. This can be done by evaluating the energy of a dimer in vacuum, systematically varying the atomic species and the radial distance between the two atoms. The result is shown in Figure S3, which demonstrates that the model captures the behavior expected of chemical bonds, showing clear repulsion at short distances, while also displaying approximately correct bond lengths for the Ti-O and Ba-O dimers.

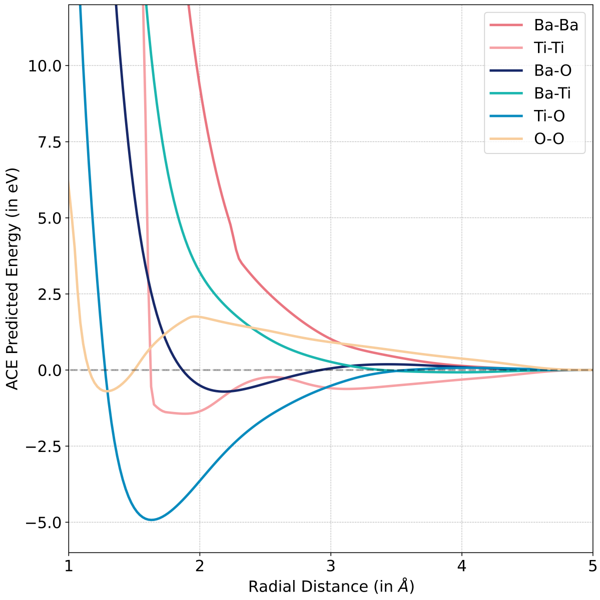

Figure S3: Energy vs radial distance plot for different dimer configurations, computed using the trained ACE potential.

S3.3 Equation of State test

To measure the efficacy of the potential for bulk systems, structures corresponding to experimentally observed phases are taken from the Materials Project, compressed and stretched by 30% to emulate volume change. The energy for each volume is computed using both the trained ACE potential and single-point DFT in VASP. A Birch-Murnaghan equation of state is fit, as shown in Figures S4. The potential displays the energy-volume well that is expected from stable crystals for all structures considered, matching well the DFT energies for most structures. Remarkably, even for structures with significant deviations in the EOS, shown in Figure S4B, the computed equilibrium volumes and bulk modulus are within 5% of those computed from DFT data. This implies that the potential has captured the curvature of the potential energy landscape well for all compositions of interest in this system, rendering it suitable for MD simulations which rely on the predicted forces rather than energies.

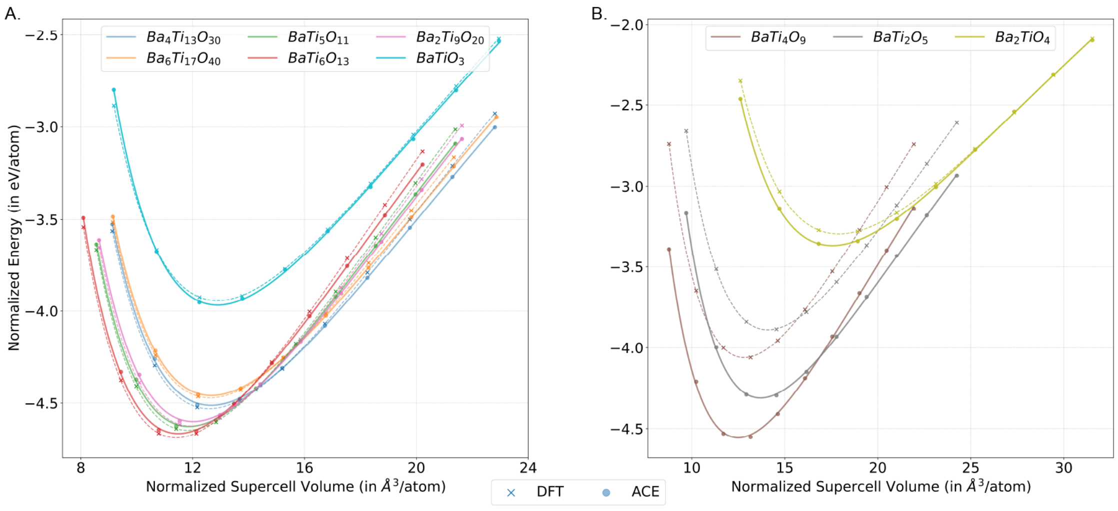

Figure S4: Energy vs Volume plots for crystalline structures taken from MP in the Ba-Ti-O system.

S3.4 RDFs and short range order

To ensure spatial correlations are properly captured, we run ACE-MD on MPMorph equilibrated structures for all compositions that were used for training the potential, and the radial distribution functions (RDFs) are computed for all pairs of atomic specie and compared to those obtained from AIMD. The RDFs for $BaTiO_3$ and Ba_2TiO_4 at 1000K are shown in Figure S5. As can be seen, the model recovers the correct short range configuration, with the position and magnitude of the first peak (i.e., the first shell) of the ACE-MD trajectory lining up almost exactly with the AIMD trajectory. There is however, a deviation after the first shell, notably for radial distances $> 5\AA$, which is likely due to the general lack of medium and long range order in these systems.

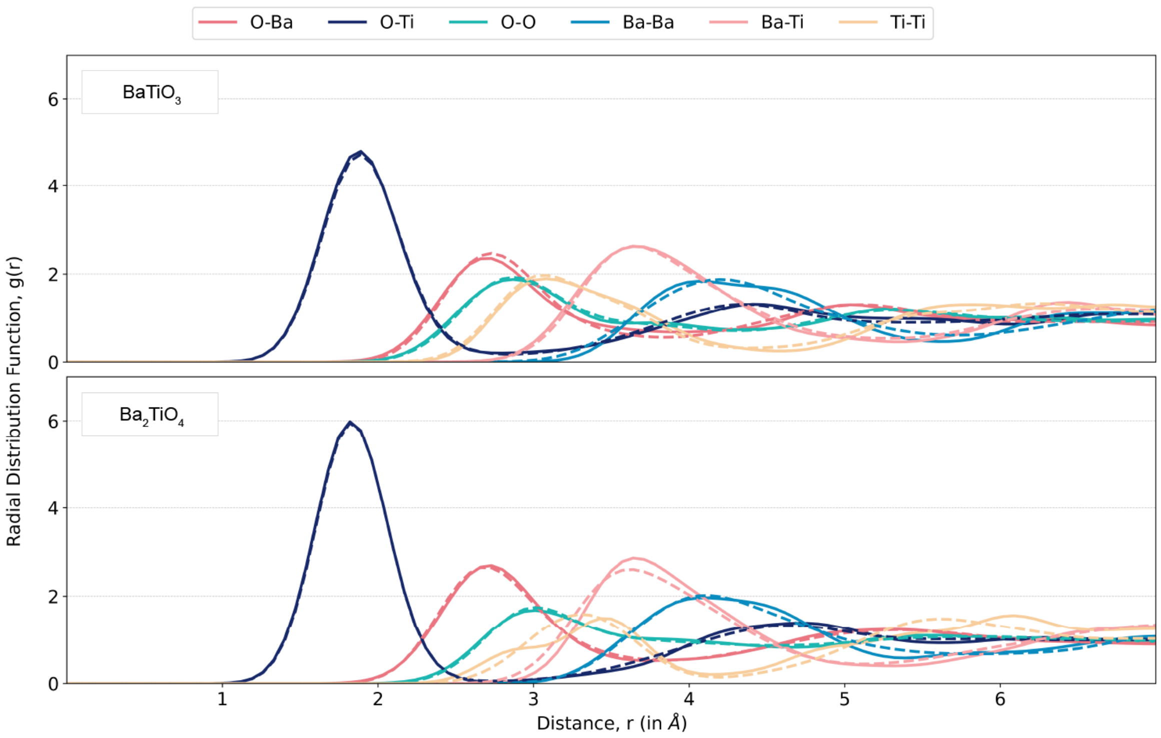

Figure S5: Comparison of RDFs obtained from ACE-MD for $BaTiO_3$ (top) and Ba_2TiO_4 (bottom), shown with the solid line, with corresponding RDFs obtained from AIMD, shown with the dashed line.

S4 Adapting Onsager transport framework to diffusive transport in amorphous solids

The Onsager transport coefficients are typically defined with respect to a reference specie in the system [1, 2]. For electrolytes, this is often the solvent being used. Due to the lack of such a reference specie, in our case we use the center of mass of the system as the reference, defining all displacements and velocities in the frame of the center of mass when computing correlation functions. This leads to an over-determined system: not all coefficients in the transport matrix are independent. By the Onsager reciprocity theorem and the second law of thermodynamics, the matrix is symmetric and positive semi-definite respectively. Further, since the reference itself depends on all other species in the system, every row and column in the transport matrix has one coefficient which is the linear combination of all other transport coefficients of that row or column:

$$\Sigma_i L_{ij} = 0 \tag{S4}$$

This equation holds due to L_{ij} being computed in the center-of-mass frame of reference, which leads to zero net mass flux in the system. For brevity, we left out two crucial constraints to the flux formulation in Equation 1 of the manuscript: linear irreversible thermodynamics assumes the presence of local thermodynamic equilibrium, and the condition of electro-neutrality. These can be stated as follows:

$$\Sigma_i c_i \nabla \mu_i = 0 \tag{S5}$$

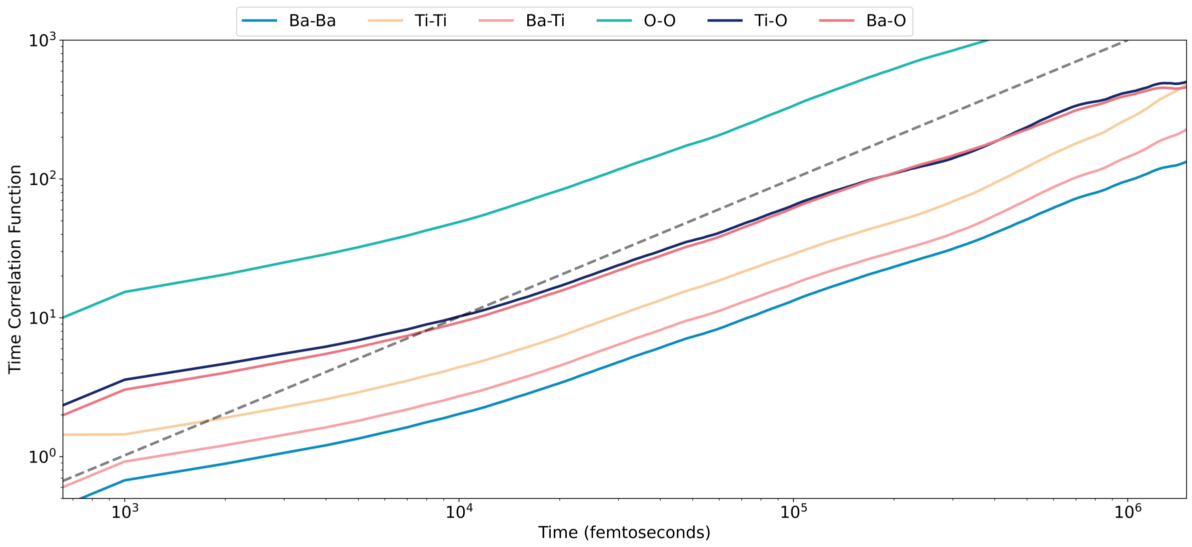

Figure S6: Displacement time-correlation function vs time plot for an ACE-MD trajectory of $BaTiO_3$ at 750K in the log-log scale. A dashed line showing a correlation function with perfectly linear slope has been provided as a guide to the eye.

$$\Sigma_i \frac{\partial(q_i c_i)}{\partial t} = 0 \tag{S6}$$

Here, q_i and c_i the charge and concentration of specie i, and $\nabla\mu_i$ is the chemical potential gradient acting on specie i. For the systems we consider in this study, $\frac{\partial(q_i c_i)}{\partial t} = 0 \; \forall$ i. Furthermore, it can be easily shown that Equations S4 and S5 constrain one variable of the system, i.e., one mass flux. These constraints allow us to exclude the O-ion flux from the definition of K_D, since this term is fixed by the definition of the Ba and Ti fluxes. We essentially consider a linear combination of the fluxes when defining the effective rate constant (K_D), thereby allowing us to capture the effect of changing driving forces and transport properties on all ions across the interface. In this work, we have three species with a first coordination shell within 3.5 $\mathring{A}$ of every atom, allowing us to calculate the ionic transport using 'smaller' systems of approximately 100 atoms. Compared to traditional self-diffusion coefficients, the Onsager transport matrix requires more statistics to fit robustly. This is primarily due to the lack of sufficient cross-ion correlation statistics (i.e., correlated movement between ions) for short timescales. Figure S6 shows the displacement time correlation function as a function of time in an ACE-MD trajectory of $BaTiO_3$ at 750K during the first 2 nanoseconds in log scale. The time scale typically achievable with AIMD would be between 10-50 picoseconds ($1 \times 10^4 - 5 \times 10^4$), where the system has not yet reached the diffusive regime (notice the difference in slopes between the computed time-correlation functions and the linear curve, shown with a dashed line). After 1.8 nanoseconds, a linear curve fit becomes feasible for all correlation functions, as their slopes align closely with the linear trend. Additional windows of linearity emerge when the simulation is extended, particularly for MD runs conducted at higher temperatures. Overall, using ACE-MD (or any MLIP-based MD) is essential for accurately fitting the full Onsager transport matrix in such ionic systems. It is worth noting that the quality of the fits for cross-coefficients is generally lower than for the self-terms. This issue could be mitigated by running ACE-MD simulations for longer durations or on larger systems. For this study, we ensured that the uncertainty

in the fit was no greater than 40% for the cross-transport terms and no greater than 20% for the net and self-transport terms. Trajectories failing to meet this criterion were excluded. Reliable fits for cross-ion coefficients were not achievable for certain phases at 750 K. Consequently, we opted to exclude data from 750 K in our analysis. The code-base for the transport coefficients can be found at `https://github.com/vir-k01/py-OATS`, and an example for such fitting is provided in the Supplementary Information files (`10.6084/m9.figshare.28207292`).

S5 Chemical Potential Diagram for Ba-Ti-O system

The chemical potential diagram at 0K as computed using `pymatgen` with all entries from the Materials Project in the Ba-Ti-O chemical space, is shown in Figure S7. In a reaction involving BaO and TiO_2, the corresponding planes are shaded red and purple, respectively. The driving force for ion movement across the reaction interface is represented by the shortest distance between these two planes, approximately indicated by the red arrow. Conversely, for a reaction between BaO and Ba_2TiO_4 (shaded green), the two planes share an edge, resulting in a shortest distance of zero. This indicates no net flux across the interface, signifying thermodynamic equilibrium. For details on how these diagrams are computed, refer to Ref [3, 4].

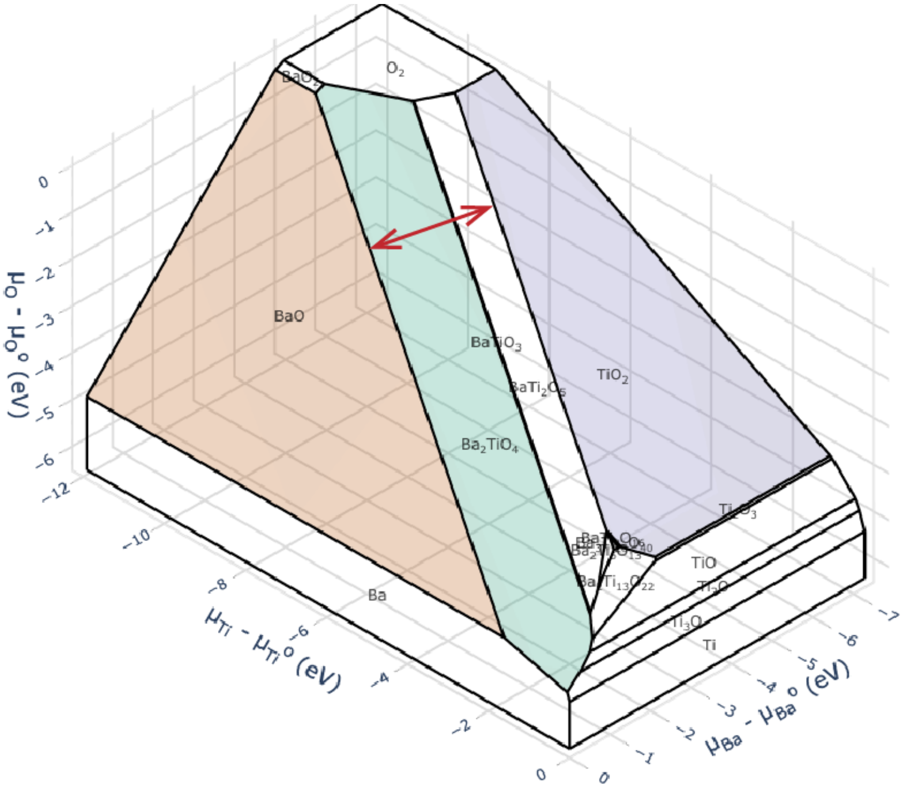

Figure S7: Chemical potential diagram for the Ba-Ti-O chemical system at 0K.

S6 Derivation of rate across a spherical powder interface

Following [5, 6], we start with the case for diffusion-limited growth in the geometry shown in Figure S8, wherein growth of a product γ happens onto a reactant particle of α. The growth rate of the

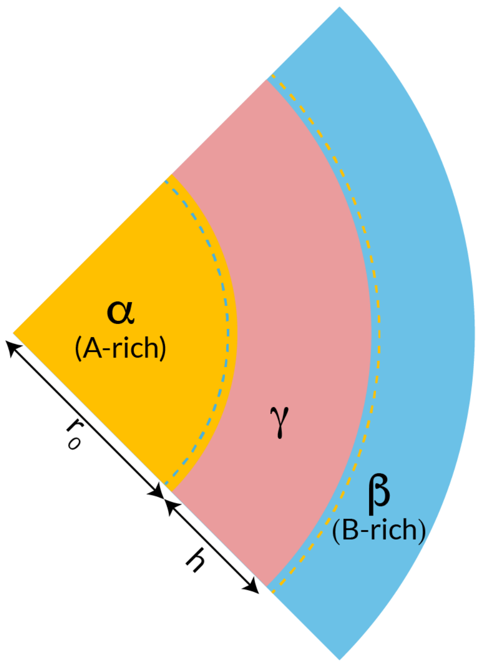

Figure S8: A schematic for the core-shell model for diffusion-controlled growth, wherein diffusion of A from α to β or B from β to α controls the rate of growth of γ. r_0 is the radius of the (spherical) particle of reactant α and h is the instantaneous thickness of the γ product.

product layer γ ($=\frac{dh}{dt}$) can be expressed as:

$$\frac{dh}{dt} = V^{\gamma}\Sigma_i \frac{|J_i^{\gamma}|}{n_i^{\gamma} N_A}$$

Here, J_i^{γ} is the number density flux of the ith ion across γ, whose transport from one reactant to the other is needed for continual growth of γ [7]. We use the fact that transport of one mole of i leads to increase of $\frac{V^{\gamma}}{n_i^{\gamma}}$ volume of the γ phase, where V^{γ}, n_i^{γ} and N_A are the molar volumes of γ, the molar fractions of specie i in γ and the Avogadro number respectively. In this work, we hypothesize that the "growth" of a nucleus of γ depends on the flux through a "liquid-like" interphase with the same composition of γ. Hence, we take J_i^{γ} as the flux through a "liquid-like" counterpart of γ, which forms at the interfaces. Here, the driving force that leads to the flux J_i^{γ} is the result of flux going into γ minus the flux going out of γ:

$$J_i^{\gamma} = J_i^{\gamma,in} + J_i^{\gamma,out} = -\Sigma_j L_{ij}(\nabla\mu_j^{in} + \nabla\mu_j^{out}) \approx -\Sigma_j L_{ij}(\frac{\mu_j^{\alpha} - \mu_j^{\beta}}{h})$$

The last step above is an approximation to allow for computing the net flux that grows γ using readily available phase diagram data from the Materials Project. Using our definition of fluxes and the effective diffusion rate constant (Equations 1, 7 of the manuscript), we can rewrite this expression as:

$$\frac{dh}{dt} = \frac{K_D}{h}$$

Assuming K_D is independent of h, we can solve this equation using the initial condition of $h(t = 0) = 0$:

$$h(t) = \sqrt{2K_D t}$$

Hence, the "rate constant" for the growth of γ is given by K_D, as defined in the main text. To align this "rate constant" to existing theories of solid-state reaction kinetics, we now define the "fraction of reactants consumed"($y(t)$) as the change in volume of the reactant α (from Figure S8):

$$y = \frac{V_\gamma}{V_\alpha} = 1 - [\frac{(r_0 - h(t))}{r_0}]^3$$

Rearranging this expression using the solution for $h(t)$ gives:

$$[1 - (1 - y(t))^{1/3}]^2 = \frac{2K_D t}{r_0}$$

This is the same form for the rate as the Jander equation[8], which has been applied extensively for the cases of diffusion-limited growth in solid-state reactions. Obviously, on repeating this exercise for the case where γ grows onto a particle of β instead, the only difference is r_0 becomes the size of the reactant β. This equivalence is because transport of all species (in this 3-component case) are inter-dependent through the Onsager definition of the flux, and we assume K_D does not depend on h or time. Hence, the rate constant K_D that we derive and compute in this work defines "selectivity" towards a product γ, and differences in K_D can be used to inform the outcomes of pair-wise powder reactions.

S7 Computed Onsager transport coefficients

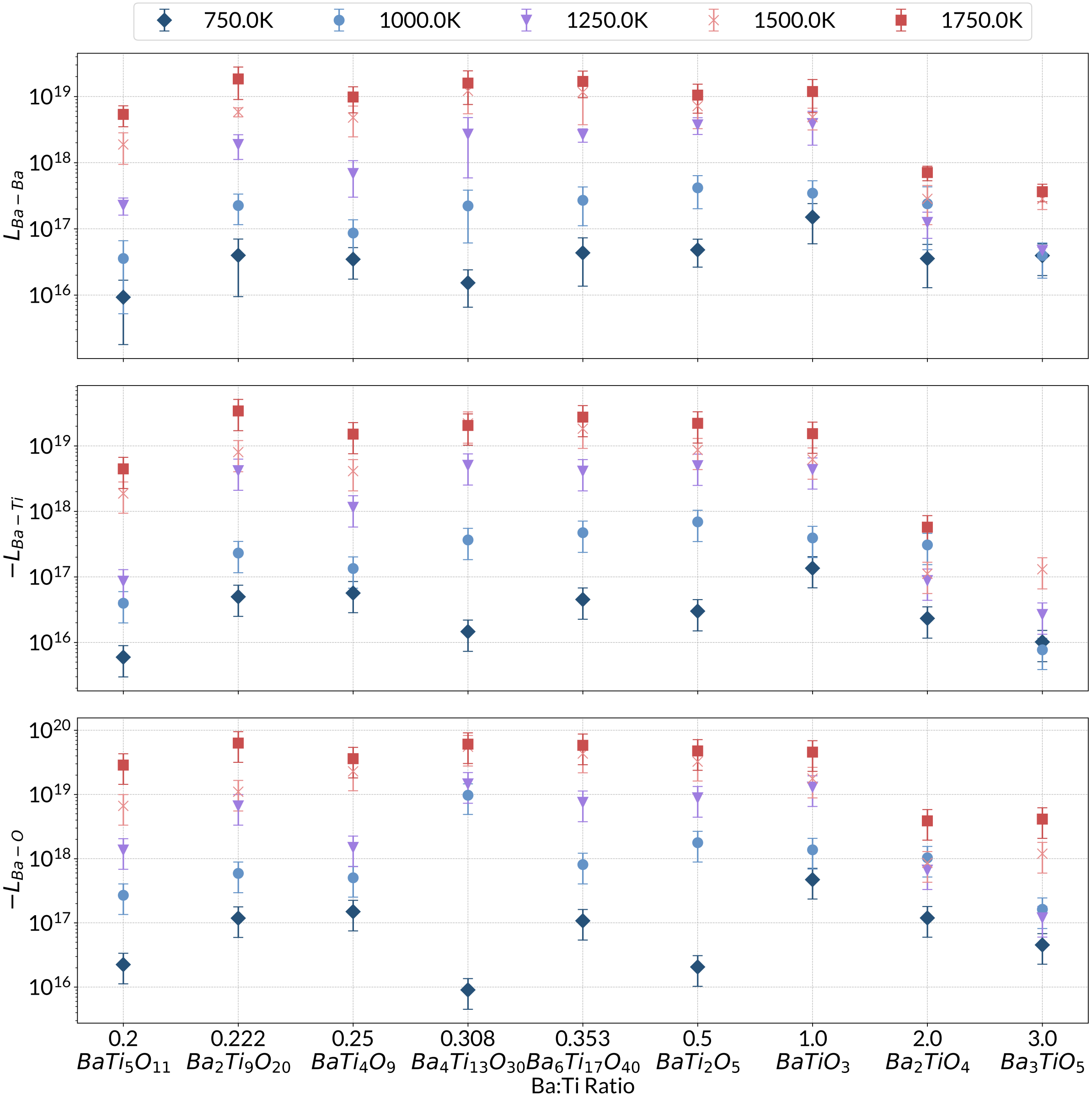

Figure S9: The computed Onsager transport coefficients (in units of $1/(eV - cm^3 - sec)$) for different Ba-Ti-O phases, ordered by increasing Ba:Ti ratio, corresponding to the Ba-Ba correlation (top), Ba-Ti correlation (middle) and Ba-O correlation (bottom). Error bars are computed using the standard deviation across 5 molecular dynamics trajectories.

S8 ReactCA simulation step and scoring parameters

For a more thorough discussion on how the ReactCA framework works, we point the reader to Gallant et. al. [9]. A birds-eye of view of a single reaction step is shown in Figure S11. We would like to point out that the reaction is selected at each step probabilistically based on scores assigned through Eq. 9 of the main text. The form of this function and the parameters used have been tuned by carefully considering their effect on reaction outcomes across several synthesis reactions, while

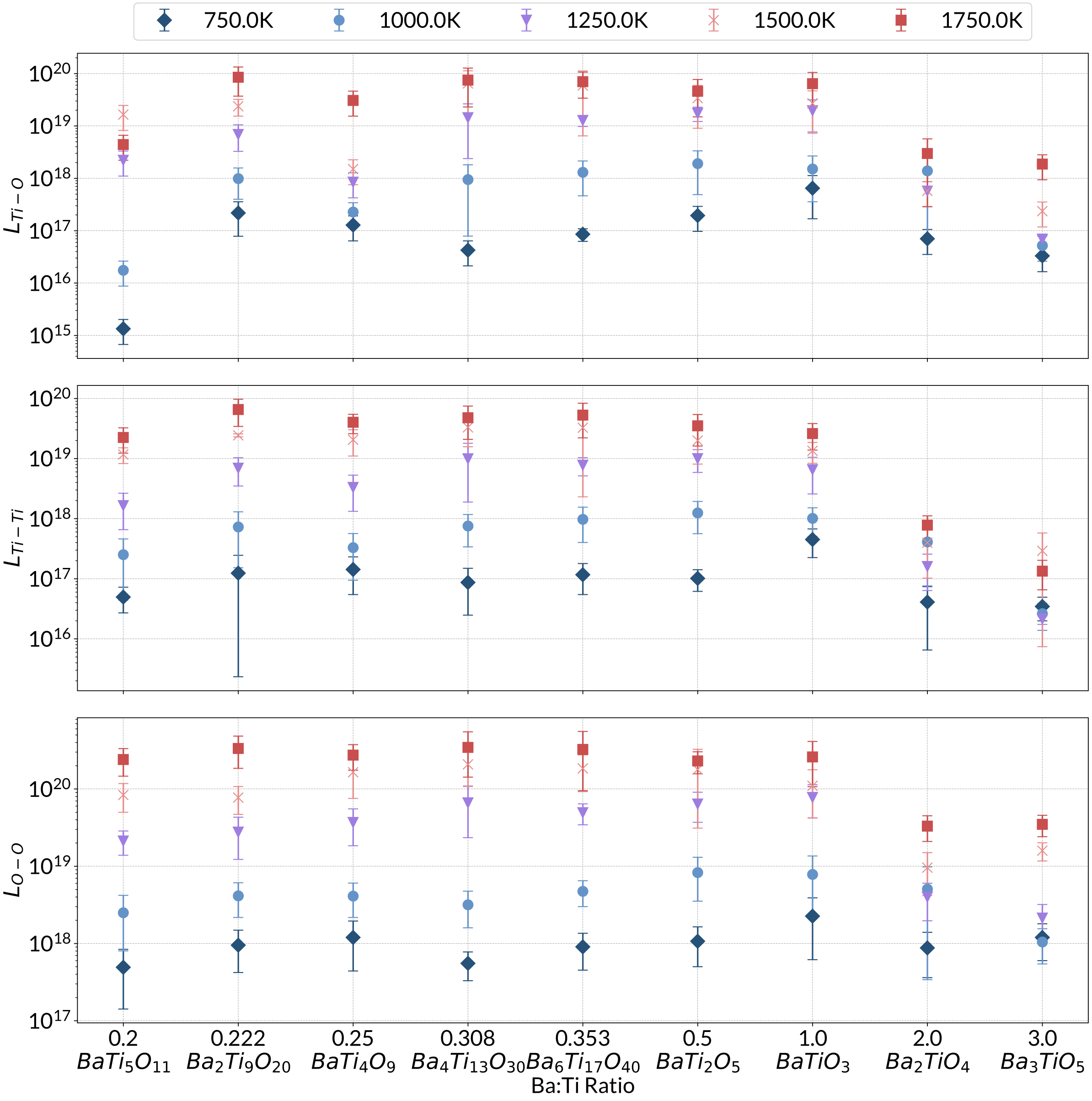

Figure S10: The computed Onsager transport coefficients (in units of $1/(eV - cm^3 - sec)$) for different Ba-Ti-O phases, ordered by increasing Ba:Ti ratio, corresponding to the Ti-O correlation (top), Ti-Ti correlation (middle) and O-O correlation (bottom). Error bars are computed using the standard deviation across 5 molecular dynamics trajectories.

trying to remain as physically sound and easy to compute as possible. An effect of the parameters that are used in this scoring function have been discussed in [9]. In this work, a new parameter: 's' is introduced to scale the kinetic contribution of the score to the Tamman's rule part of the score. To provide a basic understanding of the effect of varying 's', refer to Table S3. In general, 's' only has to be tuned once for a chemical system, and for the Ba-Ti-O case we found a value of $s = 5 \times 10^{13}$ (which leads reactions to onset $\approx 1000K$) to be satisfactory. For context, increasing 's' to 1×10^{14} decreases the onset temperature by approximately 100K and decreasing 's' to 2×10^{13}

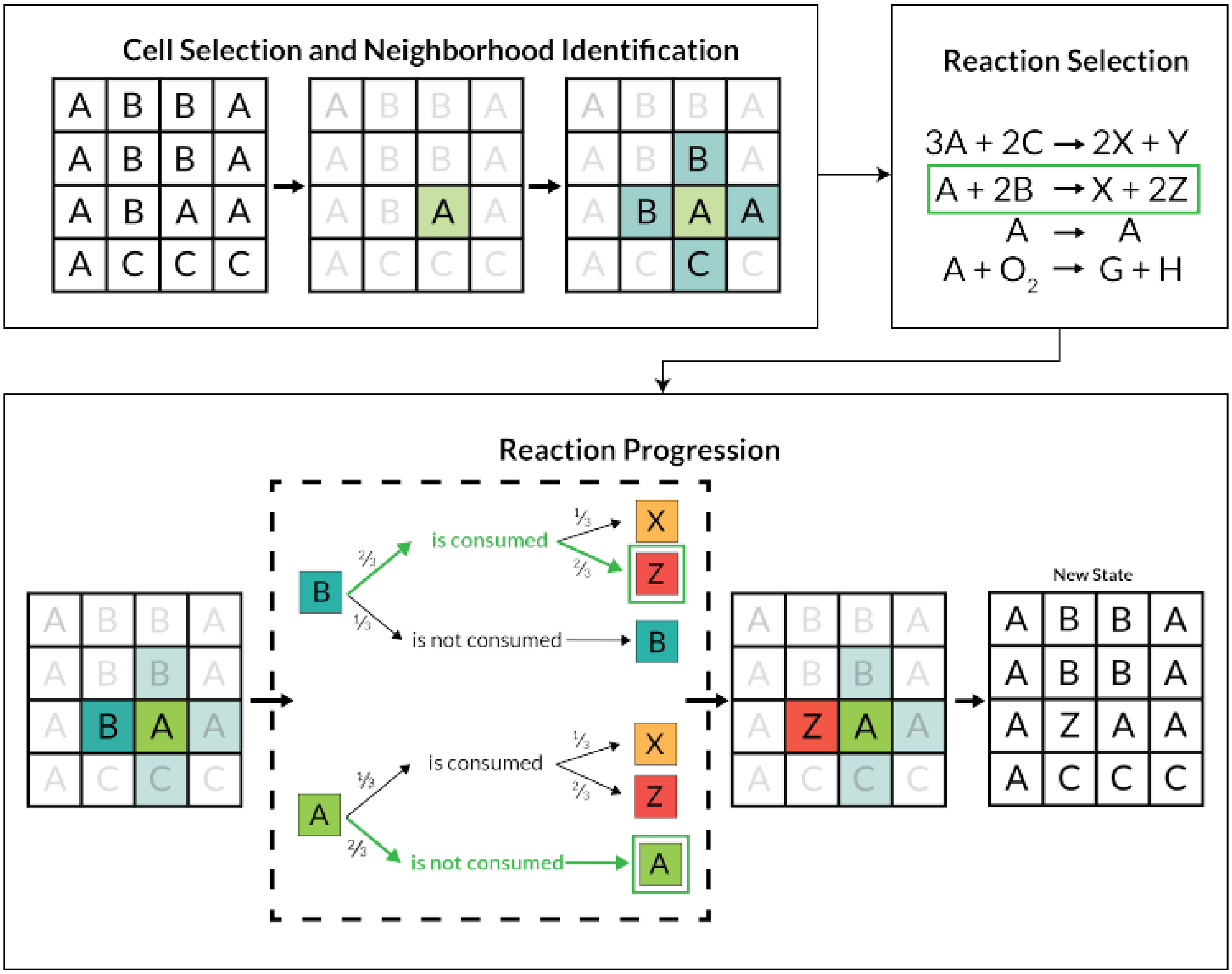

Figure S11: A single step of the ReactCA simulation. Adapted from [9].

s	$BaTiO_3$	Ba_2TiO_4	$BaTi_2O_5$	$Ba_4Ti_{13}O_{30}$
1×10^{13}	0.392	0.323	0.283	0.25
2×10^{13}	0.534	0.209	0.256	0.044
5×10^{13}	**0.932**	0.202	0.192	0.034
1×10^{14}	0.482	0.232	0.283	0.314

Table S3: Dependence of the max observed mole fractions of the phases present in Reaction 1 as a function of the scoring function parameter 's'.

increases the onset temperature by 100K. In practice, setting this parameter to be correct to within the right order of magnitude should lead to atleast qualitatively correct simulations. We only set this parameter once and use it across all the reactions studied in this work.

We acknowledge that there may be a superior form for this function that more quantitatively reflects the real reaction mechanisms at play or yields better general accuracy in predicting reaction outcomes. The development of such a scoring function constitutes a rich area of future research but will likely be most fruitful after the development of more sophisticated theories for atomistic solid-state reaction mechanisms.

References

[1] Kara D Fong et al. "Onsager transport coefficients and transference numbers in polyelectrolyte solutions and polymerized ionic liquids". In: *Macromolecules* 53.21 (2020), pp. 9503–9512.

[2] Kara D Fong et al. "Transport phenomena in electrolyte solutions: Nonequilibrium thermodynamics and statistical mechanics". In: *AIChE Journal* 66.12 (2020), e17091.

[3] H Yokokawa. "Generalized chemical potential diagram and its applications to chemical reactions at interfaces between dissimilar materials". In: *Journal of phase equilibria* 20.3 (1999), pp. 258–287.

[4] James R. Neilson, Matthew J. McDermott, and Kristin A. Persson. "Modernist Materials Synthesis: Finding Thermodynamic Shortcuts with Hyperdimensional Chemistry". en. In: *Journal of Materials Research* 38.11 (June 2023). arXiv:2303.11915 [cond-mat], pp. 2885–2893. ISSN: 0884-2914, 2044-5326. DOI: 10.1557/s43578-023-01037-2.

[5] Chung-Hsin Lu and Jiun-Ting Lee. "Kinetic analysis of the serial reactions of lead magnesium tungstate ceramics using a multiple core-shell model". In: *Journal of materials science* 33 (1998), pp. 2121–2127.

[6] FM d'Heurle. "Theoretical considerations about phase growth and phase formation". In: *MRS Online Proceedings Library* 402.1 (1995), pp. 3–14.

[7] Hermann Schmalzried. *Chemical kinetics of solids*. John Wiley & Sons, 2008.

[8] Jorge R Frade and Michael Cable. "Reexamination of the Basic Theoretical Model for the Kinetics of Solid–State Reactions". In: *Journal of the American Ceramic Society* 75.7 (1992), pp. 1949–1957.

[9] Max C. Gallant et al. "A Cellular Automaton Simulation for Predicting Phase Evolution in Solid-State Reactions". In: *Chemistry of Materials* 37.1 (2025), pp. 210–223. DOI: 10.1021/acs.chemmater.4c02301. eprint: https://doi.org/10.1021/acs.chemmater.4c02301.
